# Temperature management in central nervous infection

**DOI:** 10.1186/cc11276

**Published:** 2012-06-07

**Authors:** Erich Schmutzhard, Peter Lackner, Ronny Beer, Marlene Fischer, Anelia Dietmann, Bettina Pfausler

**Affiliations:** 1 Department of Neurology, Neurocritical Care Unit, Medical University Hospital Innsbruck, Austria

## Introduction

Brian Kellock quotes in his book *The Fibreman *the seven-year-old (that is, in the year 1918) Denis Burkitt discussing respectfully his uncle Dr Roland Burkitt's legendary reputation amongst his patients in Kenya, Africa, as: '... he believed in curing fevers, in particular in delirious patients, by artificially lowering the patients' temperature ...' [1].

Obviously, Dr Roland Burkitt, the uncle of the legendary Prof. Dr Denis Burkitt, after whom the African lymphoma and its association with Epstein-Barr virus was named, had realized that lowering the body temperature in patients with high fevers, in Kenya highly likely to being due to cerebral malaria, meningitis or encephalitis, might influence positively the course of such potentially - at this time, in many instances, definitely - life-threatening disease [1]. Zdravev stipulated that otogenous brain abscesses not only need to be surgically evacuated but, when causing cerebral herniation, may benefit from lowering body temperature. He was the first to conclude that high body temperature in patients with increased intracranial pressure may be a deleterious association [2].

The wide variety of mechanisms of injury that are exaggerated by hyperthermia and may be ameliorated by moderate hypothermia are described elsewhere in this supplement (see abstract A1). They include mechanisms of neuroexcitotoxicity [3], release of free radicals, changes in blood-brain barrier and vascular permeability, the release of proinflammatory mediators, drawing leucocytes across the blood-brain barrier, increasing the number of inflammatory cells in the brain tissue and the passage of neutrophils, phagocytes, monocytes and macrophages into the brain, additionally injuring neuronal cells by stimulating further immune reactions [4]. Whether interfering with these mechanisms by moderate hypothermia may reduce secondary insult onto neuronal cells and brain tissue is still a matter of discussion. What has been known for long time is that increased intracranial pressure, as frequently seen in viral encephalitis, severe bacterial meningitis and/or brain abscesses, may be modified by therapeutic hypothermia/targeted temperature management [5]. In addition, long-term morbidity and mortality in CNS infection may additionally deteriorate if bacterial meningitis is complicated by cerebral infarction, occurring during the course of disease [6]. Thus, preventing cerebral infarction may be an important tool in reducing both morbidity and mortality in adults with community-acquired bacterial meningitis.

Prandini and colleagues have shown that mild hypothermia reduces remarkably polymorphonuclear leucocyte infiltration in induced brain inflammation in rats compared with those without hypothermia [4].

After any type of brain injury, in particular, ischemic and ischemia reperfusion injury, the reduced brain oxygen supply quickly leads to a decrease in ATP and phosphocreatine levels, thus initiating a complex cascade of events involving excessive calcium influx into brain cells, excessive glutamate receptor activation and neuronal hyperexcitability; that is, triggering off the excitotoxic cascade [3]. Exactly this excitotoxic cascade may be responsible for provoking overt or subtle epileptic seizures in patients with CNS infection, thereby deteriorating the prognosis of these patients. Irazuzta and colleagues could nicely show that in rabbits hypothermia decreases excitatory neurotransmitter release in bacterial meningitis, in particular, the release of glutamate and aspartate, known to be involved in the pathogenesis of neuronal injury in meningitis, suggesting that hypothermia may attenuate excess neuronal stress in this disease [3].

Febrile refractory status epilepticus may be caused by presumed encephalitis carrying a very high morbidity and mortality rate [7]. In addition, it is believed that hyperthermia aggravates brain damage due to continuing epileptic activity. In a retrospective analysis Nakagawa and colleagues found a significant improvement of outcome in children with such febrile refractory status epilepticus who were treated by therapeutic hypothermia with subsequent prophylactic normothermia compared with those children with the same disease but treated only by conventional therapy without fever management [7]. The authors conclude that treatment with therapeutic hypothermia plus subsequent prophylactic normothermia may reduce neurological damage in such patients with severe acute encephalopathy due to refractory febrile convulsive status epilepticus. Another Japanese group treated an adult patient with influenza type A virus-associated encephalopathy with routine therapeutics as oseltamivir and methylprednisolone pulse therapy. Because of severe brain swelling, hypothermia was added. This combination treatment led to full recovery without neurologic sequelae [8]. A similar observation has been reported in 43 children with acute inflammatory encephalopathy and/or encephalitis, out of whom 27 were treated with mild hypothermia. These 27 children were compared with 16 similar patients who were cared for at normothermia levels [9]. The effect of therapeutic hypothermia for children with acute encephalopathy/encephalitis was observed to be dependent on the timing when cooling was initiated. Early cooling (within 12 hours of initial neurological signs of encephalopathy/encephalitis) improved outcome whereas delayed cooling (after 12 hours) tended to be even deleterious [9].

In adults, therapeutic hypothermia in CNS infection has been limited to single case reports and case series. Cuthbertson and colleagues reported induced hypothermia, together with barbiturate coma, in a meningitis patient associated with intractable raised intracranial pressure. Throughout the entire period of mild hypothermia (35°C), achieved by thiopental and external cooling, cerebral perfusion pressure was maintained above 70 mmHg. Barbiturate-induced coma and induced hypothermia were continued for 48 hours when complications (pancreatitis, ventilator-associated pneumonia) prompted one to stop these two therapy modalities. However, the patient remained without any ICP rebound, eventually being weaned from the ventilator and making a rather uneventful recovery, being discharged home with reportedly no neurologic deficits 3 weeks after the disease had began [5]. Similarly, a case with space occupying herpes simplex virus encephalitis has been reported by Wagner and colleagues. The authors correctly state that conventional intracranial pressure-lowering modalities are limited in such severe herpes simplex virus encephalitis patients and more aggressive treatment options are needed. They induced moderate hypothermia (33°C), resulting in fast and sustained control of intracranial pressure. Eventually the patient regained a relatively good functional outcome, being able to walk unassisted (GOS 4). It needs to be noted that rewarming was done very slowly (1°C/day); that is, over a period of 4 days [10].

Very recently two larger cases series have been published on the use of therapeutic hypothermia both in adult viral meningoencephalitis and in adult community-acquired meningitis by the same Croatian group:

## Therapeutic hypothermia in adult viral meningoencephalitis [11]

Eleven adult patients with viral or presumed viral encephalitis (two TBE, two HSV, one VZV, six unknown) pathogen were treated with mild hypothermia, rectal temperature aimed to be 32 to 34°C. The authors, however, do not elaborate on rewarming, speed of rewarming and any adverse effects of rewarming. Remarkably only one patient (1/11 = 9%) died, 3/11 recovered fully, 2/11 with only minor impairment and 5/11 with moderate to severe residual neurological deficit. All patients showed severely impaired GCS at admission (median GCS 8 (range 3 to 10)) and median Acute Physiology and Chronic Health Evaluation score (APACHE) being 24 (range 12 to 32). A major drawback of this study is the lack of ICP monitoring, sonographic measurement of optic nerve sheath diameter having been used as surrogate markers for ICP, both methods not being as reliable as continuous ICP and CPP monitoring. The mortality rate of 9% in this case series compared with 29% in patients with viral meningoencephalitis treated in their unit before implementation of the therapeutic hypothermia protocol. The authors suggest that in carefully selected patients, carefully regarding vasoreactivity status, and, of course, only in those with initially reduced cerebral perfusion pressure, therapeutic hypothermia may be a tool to reduce long-term morbidity and mortality.

## Therapeutic hypothermia in bacterial meningitis [12]

The same Croatian group presented a series of 10 patients with severe bacterial meningitis (nine patients: pneumococci, one patient: *Escherichia coli*) with an initial median GCS of 6 (range 3 to 9), APACHE II ranging from 22 to 34 (median 31). They employed the same protocol of non-invasive ICP monitoring (using transcranial Doppler sonography, optic nerve sheath diameter sonography and jugular bulb oximetry). Hypothermia was induced by intravenous infusion of cold isotonic saline and maintained with continuous venovenous hemofiltration at 32 to 34°C. Nothing is said about rewarming, rewarming speed and duration of hypothermia. Two patients died within 48 hours from admission because of refractory intracranial hypertension, two more patients with severe residual neurological deficits (GOS 2) died later on, after discharge from the ICU, because of late-onset nosocomial sepsis; in total, a rather high mortality rate [13,14]. The surviving six patients had a mean ICU stay of 22 days (range 8 to 36), two had a severe and two a moderate residual neurologic deficit. Two of the entire group of 10 patients with bacterial meningitis had a complete neurological recovery (GOS 5).

The authors discuss hypothermia as a neuroprotective measure in severe bacterial meningitis. In nine (out of 10) patients pneumococci were isolated as the causative organisms; however, the authors do not elaborate whether these patients - qualifying early for dexamethasone treatment, in particular, since they showed severe clinical signs and symptoms of increased intracranial pressure - had been given dexamethasone or not. They discuss that corticosteroids have not substantially changed disease outcomes among patients with most severe pneumococcal meningitis. Exactly this aspect is in contrast to the widely accepted knowledge and level of evidence respectively. Recently a meta-analysis has clearly shown that European patients with pneumococcal bacterial meningitis (aged >55 years) definitely benefit from the adjunctive application of dexamethasone [15]. Only one single patient would not have qualified for this definition (47-year-old female being severely immunocompromised). This might explain the high mortality rate despite the administration of the adjunctive therapy with moderate hypothermia. The authors correctly claim that any adjunctive therapy should be continued beyond the very first hours and days since it has been known that in severe bacterial meningitis vasculopathy leading to ischemic stroke may ensue even days after onset. Adequate maintenance of sufficient cerebral perfusion pressure until recovery of CO_2 _reactivity (not to be expected before day 4 after initiation of antibiotic treatment) is essential and requires therapeutic hypothermia to be maintained for this period of time. However, the results of this pilot trial in 10 consecutive patients do not justify one to recommend therapeutic hypothermia as adjunctive therapy in bacterial (pneumococcal) meningitis. Whether selected patients with either intractable ICP, dangerously low CPP or infection-associated refractory status epilepticus benefit from moderate hypothermia needs to be elucidated; today this is still a case to case decision.
